# Cranial trephination and infectious disease in the Eastern Mediterranean: The evidence from two elite brothers from Late Bronze Megiddo, Israel

**DOI:** 10.1371/journal.pone.0281020

**Published:** 2023-02-22

**Authors:** Rachel Kalisher, Melissa S. Cradic, Matthew J. Adams, Mario A. S. Martin, Israel Finkelstein

**Affiliations:** 1 Joukowsky Institute for Archaeology and the Ancient World, Brown University, Providence, Rhode Island, United States of America; 2 Department of History, University at Albany, State University of New York, Albany, New York, United States of America; 3 W.F. Albright Institute for Archaeological Research, Jerusalem, Israel; 4 Leon Recanati Institute for Maritime Studies, University of Haifa, Haifa, Israel; 5 Institute of Ancient History and Ancient Near Eastern Studies, University of Innsbruck, Vienna, Austria; 6 School of Archaeology and Maritime Cultures, University of Haifa, Haifa, Israel; University of California Santa Cruz, UNITED STATES

## Abstract

Here we present the paleopathological profiles of two young adult males, identified as brothers through ancient DNA analysis, who were buried together beneath the floor of an elite early Late Bronze Age I (ca. 1550–1450 BC) domestic structure at the urban center of Megiddo (modern Israel). Both individuals displayed uncommon morphological variants related to developmental conditions, and each exhibited extensive bone remodeling consistent with chronic infectious disease. Additionally, one brother had a healed fracture of the nose, as well as a large square piece of bone cut from the frontal bone (cranial trephination). We consider the potential etiologies for the appearance of the skeletal anomalies and lesions. Based on the bioarchaeological context, we propose that a shared epigenetic landscape predisposed the brothers to acquiring an infectious disease and their elite status privileged them enough to endure it. We then contextualize these potential illnesses and disorders with the trephination procedure. The infrequency of trephination in the region indicates that only selected individuals could access such a procedure, and the severity of the pathological lesions suggests the procedure was possibly intended as curative to deteriorating health. Ultimately, both brothers were buried with the same rites as others in their community, thus demonstrating their continued integration in society even after death.

## Introduction

### The site of Megiddo

Tel Megiddo is one of the most important archaeological sites in the ancient Near East. Located in the Jezreel Valley (modern Israel), in the past Megiddo stood at and controlled part of the Via Maris, an important land route that connected Egypt with Syria-Mesopotamia and Anatolia [1–3]. The settlement’s prominent location played an important role in its development as a wealthy urban center during the Middle and Late Bronze Ages (ca. 1950–1130 BC). During these periods, the city reached its zenith of size, population, and wealth, with investments in monumental architecture including palaces, temples, fortifications and gates. As evidence of its status, Megiddo is documented in the royal Late Bronze Age Amarna Letters, an archive of diplomatic correspondence between Egypt and its vassals from the 14^th^ century BC [4].

**Fig 1:**
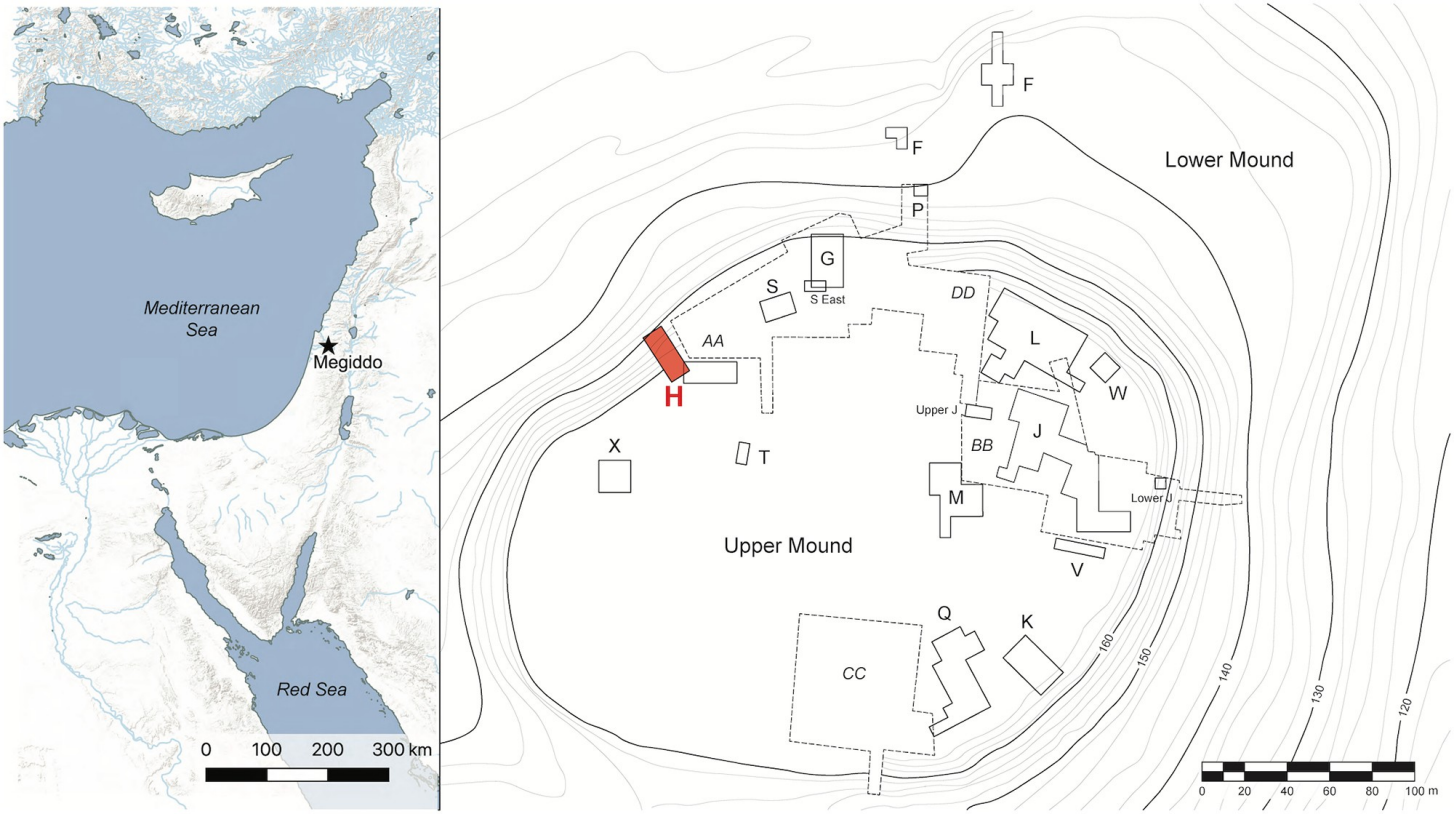
Map of the southern Levant and the site of Megiddo. On the left, the location of Megiddo in the southern Levant (Esri ArcGIS Pro, and Ancient World Mapping Center, “Waterway”; “Coastline” http://awmc.unc.edu/wordpress/map-files/ [Accessed: June 10, 2022], used under ODbL 1.0 and DbCL 1.0 licenses). On the right, the excavated areas at Megiddo, with Area H highlighted in red (plan courtesy of the Megiddo Expedition and presented here under a CC BY license).

The city’s Bronze Age palace (excavated by a University of Chicago team in the 1930s in Area AA) was erected in the northwest of the city [5, 6], approximately 15–20 m to the east of a domestic structure known as Area H (Fig 1). The proximity of Area H to the palace, in combination with its sizeable assemblage of imported Cypriot pottery, other fine wares, and precious materials, may indicate that this building was part of the palace complex, possibly functioning as an annex or an elite residence for attached officials [7, 8].

### Archaeological context of Tomb 45

**Fig 2:**
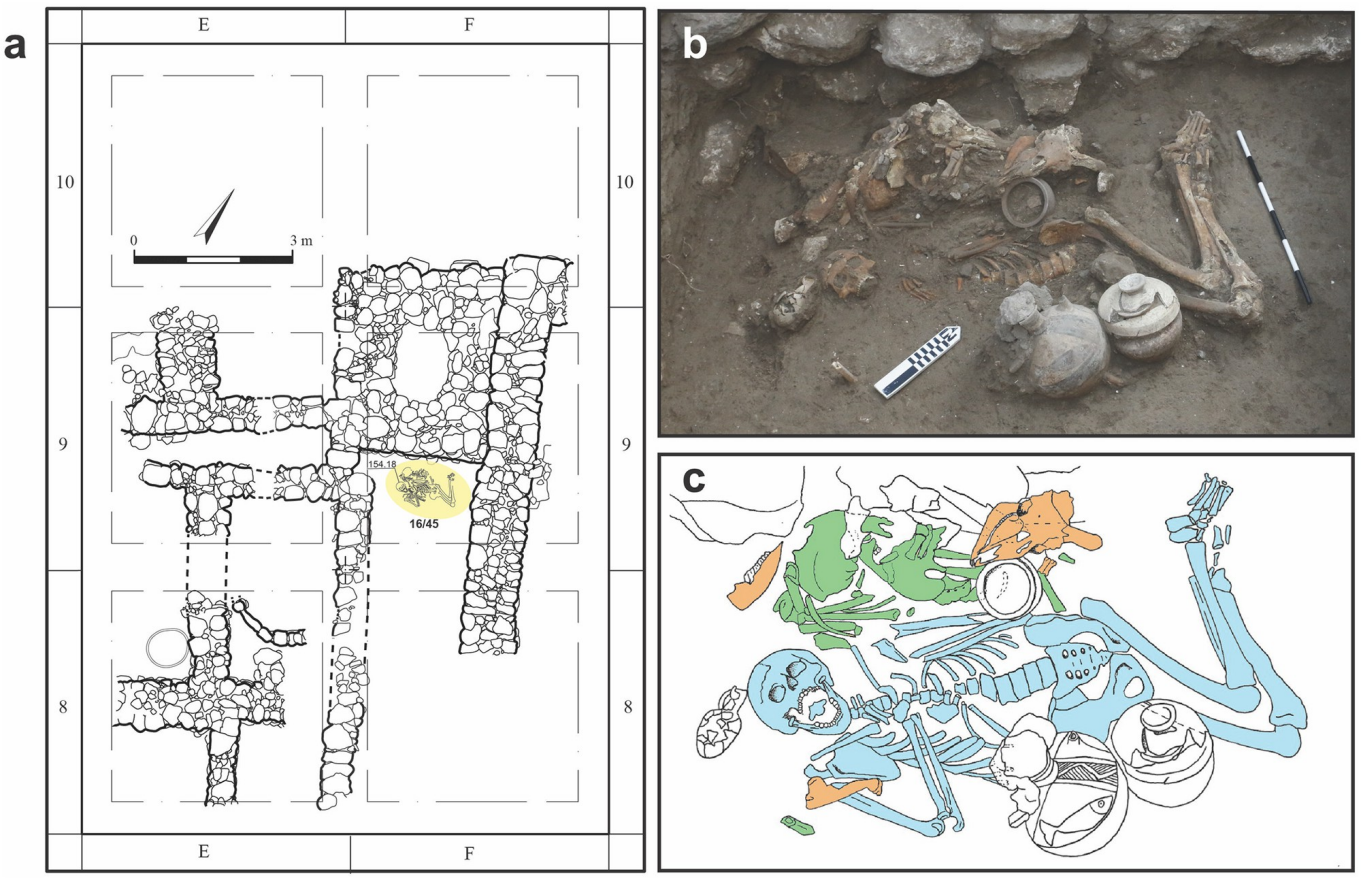
Bioarchaeological context of this study. a: The Area H (H-15) domestic structure, with Tomb 45 highlighted in yellow. (Adapted from [7] under a CC BY license, with permission from the Megiddo Expedition, original copyright 2022). b: In-situ photograph of early exposure of burial context. c: Composite drawing featuring all layers. Individual 1 is blue, Individual 2 is green, faunal remains are orange. Note the fragment of Individual 2 to the right of Individual 1’s right humerus.

During the 2016 field season, Area H yielded two subfloor tomb contexts. The first was Tomb 50, a royal Middle Bronze Age III (ca. 1650–1550 BC; Level H-16) built chamber tomb containing minimally 17 individuals adorned with gold, silver, and bronze jewelry, and afforded with dozens of ceramic vessels, quality food offerings, and incised bone inlays [8, 9]. The second tomb, located 1.75 m north of and one level above Tomb 50, was an elite Late Bronze I (ca. 1550–1450 BC; Level H-15) simple pit burial, labeled Tomb 45, which contained two adult males buried with similar quality food offerings, and fine ceramic vessels (Fig 2, S1 Text). While separated in both vertical and horizontal space, the proximity between Tomb 45 and Tomb 50 supports the notion that the later inhumation may have been intentionally placed in the vicinity of the earlier chamber tomb to assert a connection with the past [8]. Domestic subfloor burial was still in use the time of Tomb 45, a tradition that commenced in the Middle Bronze Age [10]. Its emergence has been interpreted as a marked shift to a kin-based funerary custom; one where families could tend to and care for the dead with increased intimacy [11–14]. At Megiddo (and elsewhere in the region), this burial practice came to an end by the subsequent Late Bronze IIA (starting c. 1400 BC) period [15].

Here we focus on the two individuals from Tomb 45, determined to be brothers through a previous ancient DNA study (Sample IDs I10769 and I10770) [16]. Individual 1 was in primary articulation while Individual 2 was represented by a concentrated deposit of mostly disarticulated bones, topped by caprid remains. Given that this burial was a filled, simple pit, the reconstruction of events suggests that Individual 2 was originally buried in the pit, excavated, gathered, and re-buried alongside Individual 1. Moving decomposed remains to the periphery of a burial for another inhumation was a common practice in the southern Levant in the Middle and Late Bronze Ages [17]. Therefore, the sequence of events in Burial 45 indicates that Individual 2 died and was buried shortly (c. 1–3 years) before Individual 1’s death and burial (S1 Text). In the following section, we discuss the osteological findings from individuals in the order they were identified, rather than their sequence of deposition.

## Osteological summaries of Individuals 1 and 2

### Individual 1

#### Basic demographic profile

Individual 1 was a male aged 21–46 years based on the appearance of the pubic symphysis [18], with an estimated stature of 168.77 ± 3.94 cm [19]. On the orbits were healed lesions consistent with *cribra orbitalia*, a commonly encountered, nonspecific skeletal manifestation most frequently interpreted as a sign of anemia or nutritional deficiency in childhood [20]. All 32 teeth plus a supernumerary molar were recovered (see below). Four teeth exhibit slight linear enamel hypoplasias (LEH), which reflect arrest of enamel growth during periods of stress. None of the teeth have dental caries, and most of the maxillary teeth have accumulations of dental calculus.

#### Congenital anomalies

This individual demonstrates a persistent metopic suture, an osteological variant that can persist throughout adulthood without manifesting any clinical symptoms aside from occasionally impacting the size and shape of the frontal sinuses [21]. Like other skeletal morphologies, metopic sutures vary in frequency at the population level. For example, metopism frequency in modern Europeans fluctuates between 0.0–13.0% [21], while among modern Bedouins it is more frequent, observed in 17.0–23.0% of the population [22, 23]. Metopism is also relatively common in some ancient contexts. Three of 27 (11.1%) observed crania from Middle Bronze Age Jericho exhibit metopic sutures [24]. At Iron Age Lachish, 26 of 341 (7.6%) adult males and 22 of 267 (8.2%) adult females were metopic [25]. Contemporary Iron Age Egyptian groups show lower frequencies of metopism. For example, at ancient Kerma in Nubia (modern Sudan), 4.5% males and 6.2% females had metopism, and at Sedmet, 4.3% of pooled males and females displayed the trait [25]. But at Megiddo, to date, there are no other contemporary individuals analyzed and published that display the trait. Metopism has been observed on only one Early Bronze and two Iron Age individuals from Megiddo [26]. While the abbreviated nature of past Megiddo anthropological reports makes likely that this trait is under published, the presence of this suture is infrequent enough that it is likely idiosyncratic and not necessarily representative of a population-specific trait.

**Fig 3:**
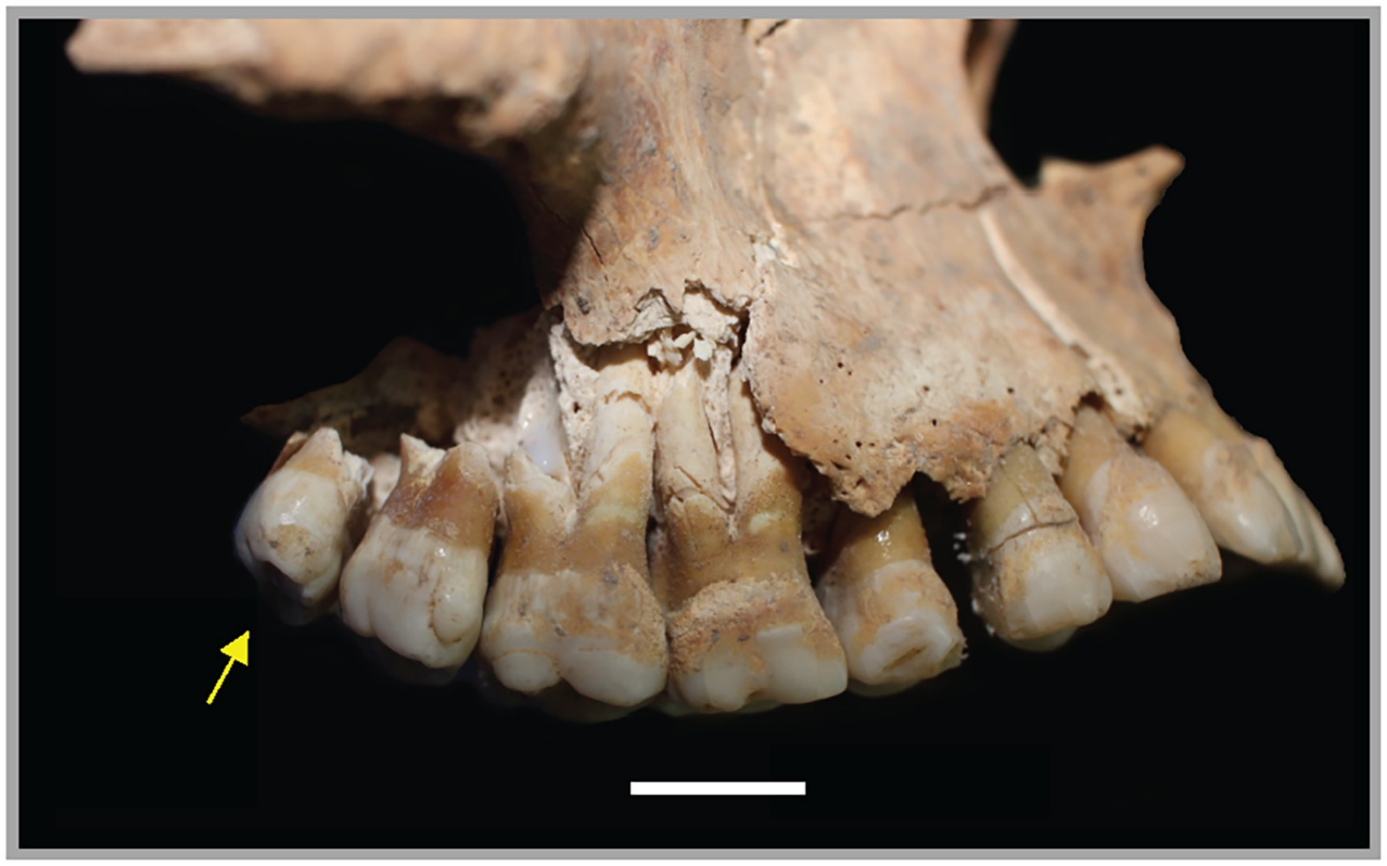
Fourth molar of Individual 1. Image shown in right lateral view. Scale bar is 1 cm.

An additional congenital condition observed was the presence of a fourth right maxillary molar (Fig 3), which is even less common than a persistent metopic suture, occurring in 1.5–3.5% of global modern adult dentitions [27], with an average of about 2.6% [28]. Supernumerary teeth reflect the hyperactivity of dental germs during growth; they can erupt anywhere in the dental arcade, and can be amorphous in shape [29]. However, the fourth molar of Individual 1 maintained morphology similar to that of the maxillary third molars. There are no fourth molars in the other quadrants. In our review of the extant literature, there are no mentions of supernumerary teeth in contemporary corpora in the southern Levant except for Iron Age Lachish, where two crania had this trait: one in the anterior arcade and the other in the middle of the palate [25].

#### Infectious disease

**Fig 4:**
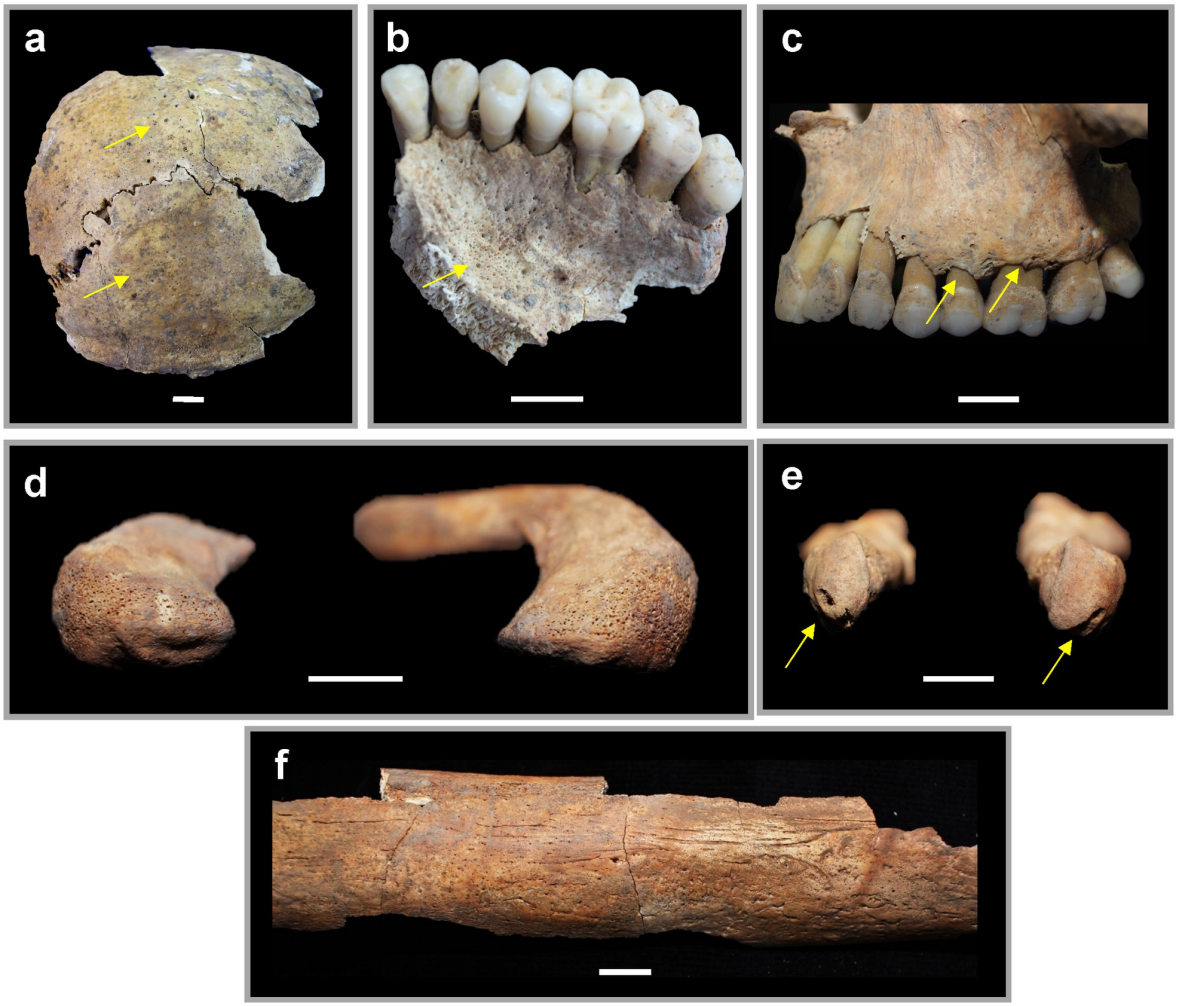
Selected lesions and anomalies of Individual 1. a: Posterior cranium with arrows pointing towards increased porosity. b: Left maxilla in inferior view with arrows highlighting the increased porosity of the hard palate. c: Left maxilla in lateral view with arrows showing alveolar recession. d: Osteolytic acromial ends of both clavicles in lateral view. e: Distal ends of both fifth metatarsals showing compressed heads with arrows pointing at the circular indentations, and f: Left humerus at midshaft with porosity and sclerotic periosteal impressions. All scale bars are 1 cm.

In addition to congenital anomalies, signs of acquired disease were present in Individual 1, some of which are shown in Fig 4. The extensive pathologies of the cranial and postcranial skeleton, many of which were bilateral, suggest that the infection or other acquired disease was systemic. Approximately one third of the recovered skeleton displays porosity (osteolytic lesions), periosteal lesions and sclerotic (new ossification) periostosis. While porosity can be a normal variant in otherwise healthy individuals, its extensiveness in Individual 1, alongside other forms of lesions, indicates sustained illness. Beginning with the cranium, the superior and posterior aspects of the cranial vault exhibit marked porosity (Fig 4a). There is also recession of the alveolus and porosity on the hard palate of the maxillae (Fig 4b and 4c). Osteolytic lesions are evident on both acromial ends (but neither sternal end) of the clavicles (Fig 4d), which potentially impacted joint mobility of the shoulder. A porotic appearance similar to the clavicles was found on the distal left ulna and the anterior sacrum. The left humerus had significant sclerotic periostosis marked by thick, smooth bone through which numerous vessel impressions were visible. Finally, the distal head of the right first metacarpal is flattened, and the distal heads of both fifth metatarsals are mediolaterally compressed and indented on the articular surface (Fig 4e; S5 Fig). This condition was not observed on other metatarsals.

#### Trauma

**Fig 5:**
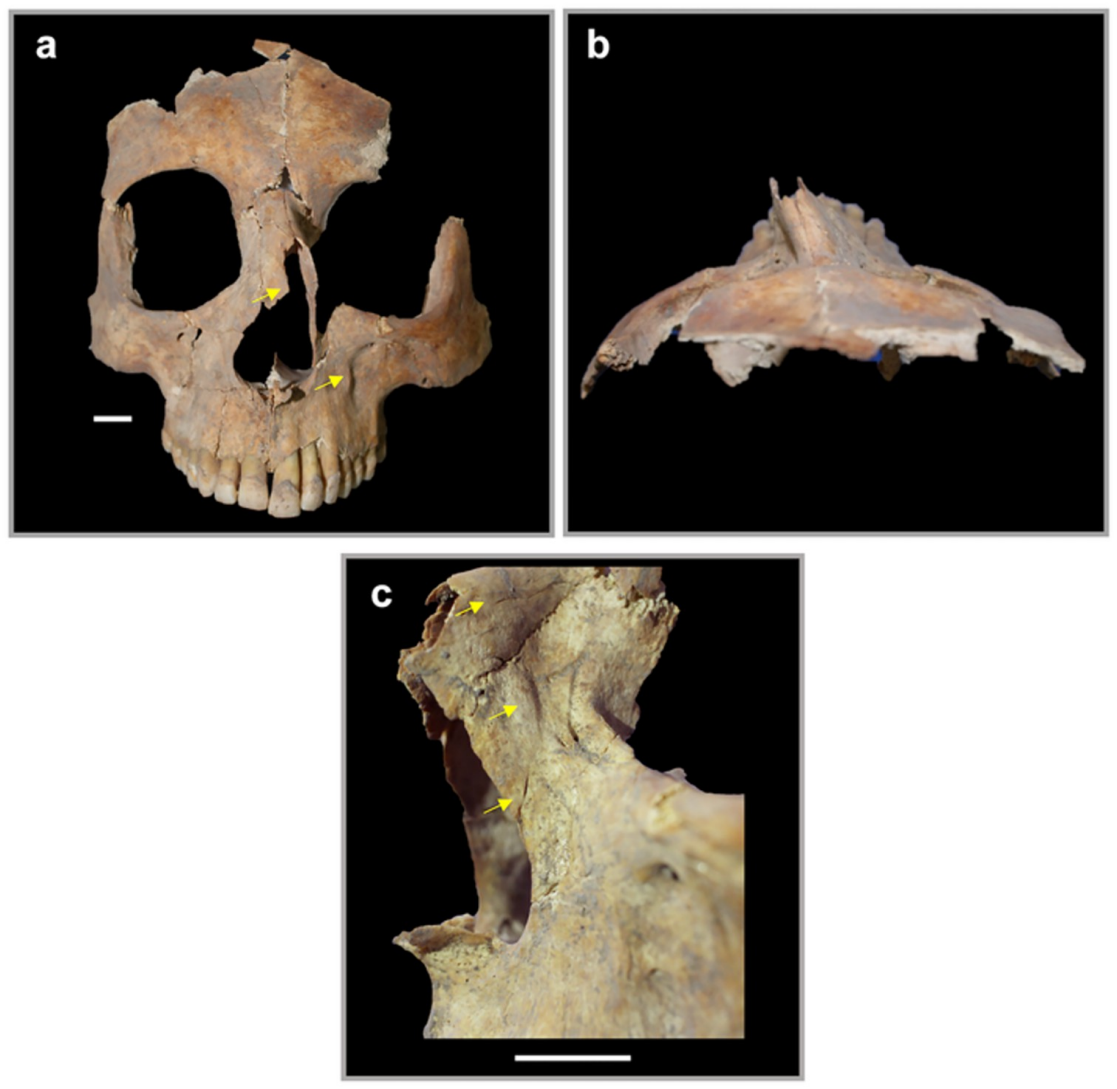
Facial trauma of Individual 1. a: Asymmetry of nasal region in anterior view, with arrows highlighting the abnormal shape of the anterior right nasal and the depression in the left maxilla. b: Asymmetry of nasals from superior view. c: The left maxilla in lateral view, with arrows highlighting healed fractures (top and bottom arrows) and the swelling of bone (center arrow). All scale bars are 1 cm.

The nasal region of Individual 1 is misshapen. Both nasal bones are asymmetrical, leaning severely to the left, with the anterior-most edge of the right nasal remodeled. Additionally, there is a large depression in the infraorbital region of the left maxilla (Fig 5a and 5b). There are three points of interest on the left nasal area (Fig 5c). Most superior is a probable fracture on the left nasal, most inferior is a healed fracture on the left maxilla, and in between them is a swelling of bone. The chain of events that resulted in the nasal asymmetry is unclear. One possible scenario is that a significant traumatic event occurred years prior to Individual 1’s death, the wound was never properly set, and resulted in the complete reshaping of the central facial region. Another possibility is that the nasal asymmetry existed prior to the fracture. Asymmetrical nasals have been observed in individuals with Goltz’s syndrome [30] and Branchio-Oculo-Facial syndrome [31], although it is worth noting that Individual 1 shows no other traits typical to these conditions. Nevertheless, if asymmetry predates the fractures, then the traumatic event may have been more readily sustained on a compromised nasal structure.

#### Trephination

**Fig 6:**
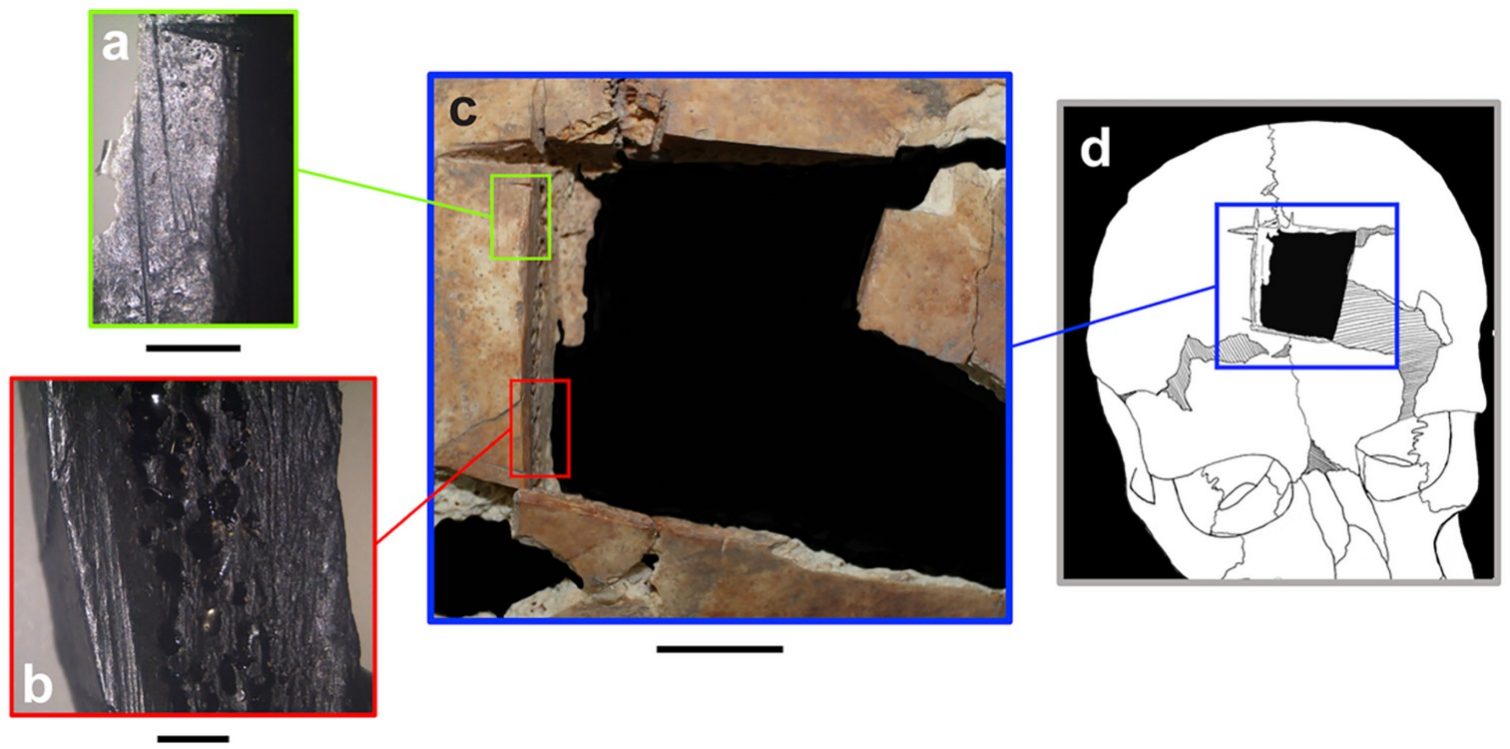
Trephination of Individual 1. a-b: Magnified edges of the trephination, each with a 2 mm scale bar. Images captured with a Leica EZ4D stereo microscope. c: All four edges of the trephination, scale bar is 1 cm. d: Reconstructed location of trephination on head.

Finally, Individual 1 had a large square piece of bone removed from the midline frontal bone, atop the persistent metopic suture. The hole measures 32 x 31 mm at its widest point and was formed by a series of intersecting notches at each corner ranging between 80° and 101°. The instrument used for the procedure appears to have been fine with a sharp beveled edge, leaving clean margins (Fig 6a and 6b). A small portion of the inner cranial table was still preserved on the upper-right edge (Fig 6c and 6d). Accompanying the trephination are smaller longitudinal scratches consistent with the opening of the scalp, a necessary step prior to bone excision [32]. We classify this as a trephination, which is defined as the removal of a piece of skull of a living individual without affecting the underlying soft tissue [33, 34].

The trephination is considered a perimortem event because 1) the color and smooth beveling of the opening’s margins indicate that the cut was made into living bone, 2) the remnant inner table of cranial bone suggests that care was taken to not puncture the underlying dura mater, which would matter only if the individual was alive, and 3) there is no evidence for postoperative bone growth. Studies demonstrate that early signs of healing are not discernable microscopically until one to two weeks post-insult [35] and macroscopic changes (such as the rounding of the edges and bone coalescence) do not begin until two to five months post operation [33, 34, 36]. As this individual exhibited neither micro- nor macroscopic changes, we suggest a reconstruction where they died either during or within a week of the procedure.

**Fig 7:**
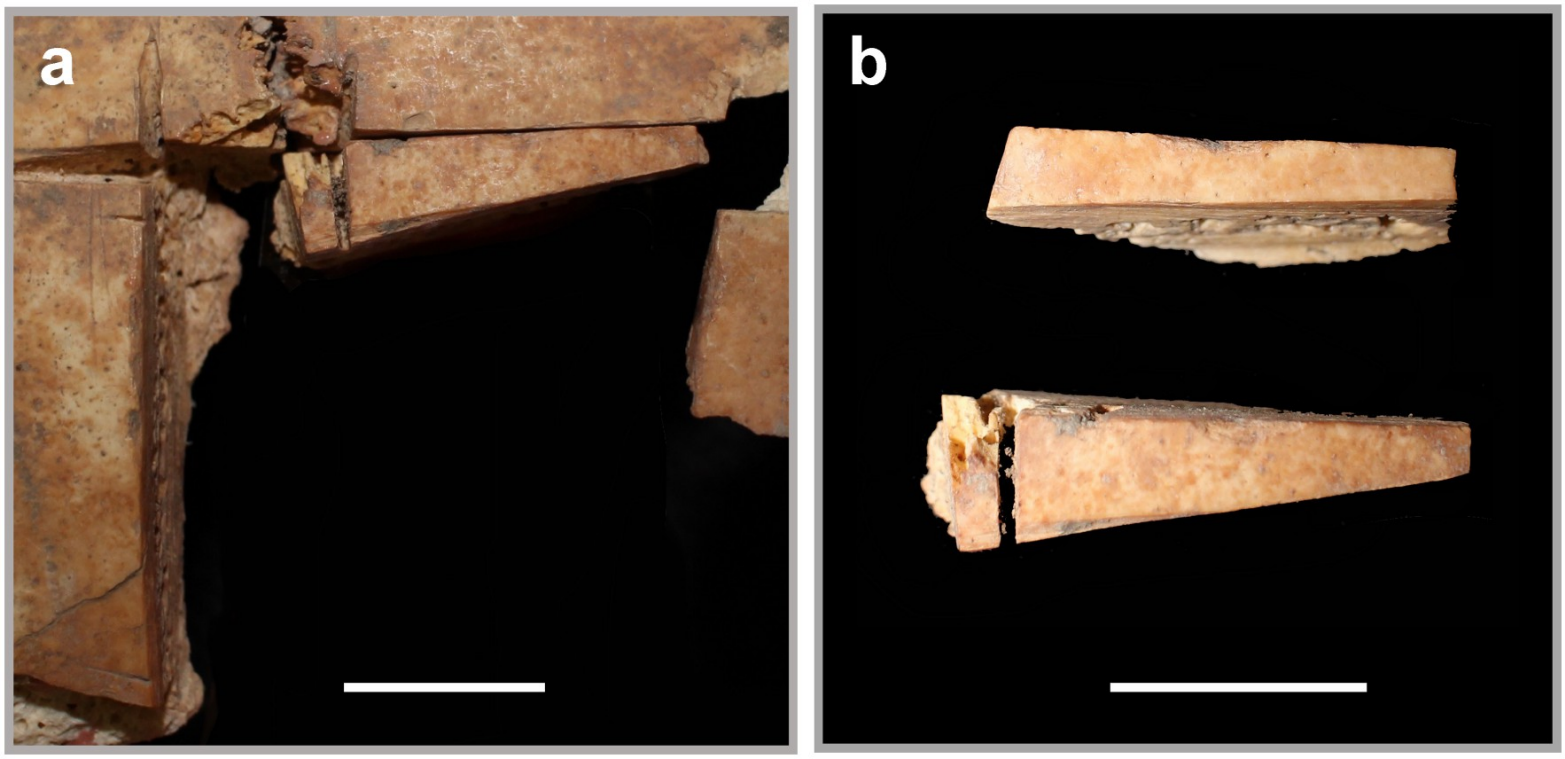
Excised cranial pieces. Left: Trephination with refit excised cranial piece. Right: Both extant pieces found during analysis. All scale bars are 1 cm.

Two excised cranial pieces were identified during analysis (Fig 7); one was collected with the cranial fragments and the other was stored with bone varia from sifted sediment. Each piece was cut on at least two sides, indicating that the trephination was done piecemeal. This process reinforces descriptions given by Malbot and Vernau [37] and later, Ferembach and Dothan [38], who hypothesized that angular notched trephinations were done by first removing a flap of skin and then making one to two initial grooves. In their model, practitioners stopped grooving when they reached the inner table of cranial bone in order to avoid the soft tissues immediately beneath it. When all grooved pieces were mostly mobile, the practitioner used leverage to remove them all at once. In most cases, the operators avoided the bone adjacent to the sutures. The Megiddo example is explained well by this general model for angular notched trephination.

### Individual 2

#### Basic demographic profile

Individual 2 is a young adult male aged in his late teens to early twenties based on the lack of fusion of the medial epiphyses of the clavicles [39, 40]. Like his brother, minor signs of healed *cribra orbitalia* were also present in Individual 2.

#### Congenital anomalies

**Fig 8:**
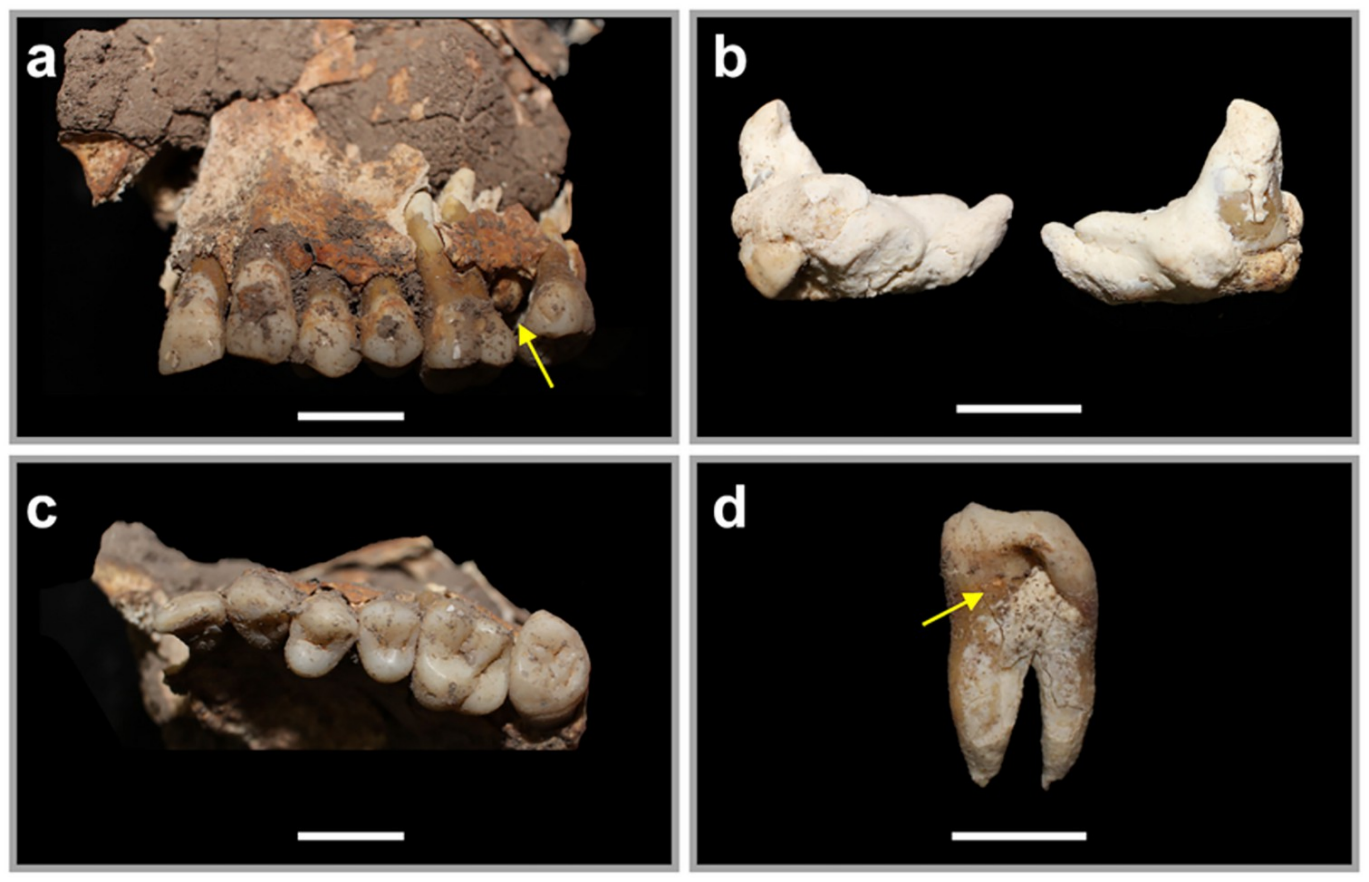
The dental anomalies of Individual 2. a: Labial view of the left maxilla with arrow showing odontogenic cyst *in-situ*. b: labial and lingual views of the odontogenic cyst. c: occlusal view of the left maxilla. d: the upper left third molar indented from the unerupted growth in mesial view. All scale bars are 1cm.

Within the crypt of the left maxillary molars was a calcified odontogenic cyst (Fig 8a and 8b), which appears to be the unerupted second molar that accumulated cementum and occupied space within the maxilla. In its absence, the third molar erupted into full occlusion, taking the position of the second molar, with the mesial surface indented by the odontogenic cyst (Fig 8c and 8d). This was the only developmental abnormality observed in Individual 2.

#### Infectious disease

**Fig 9:**
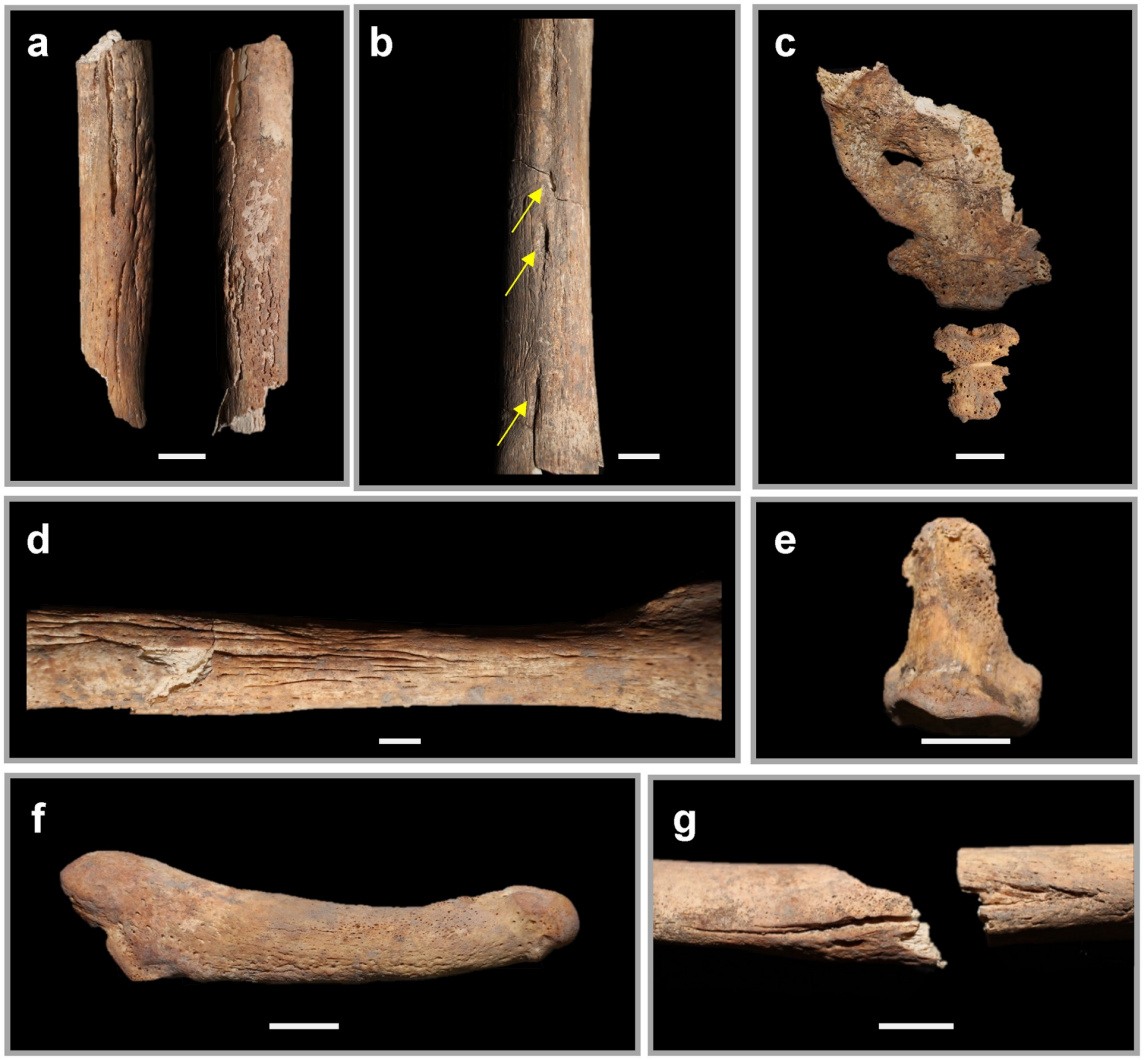
Selected skeletal elements from Individual 2 which show the extent and variety of the lesions. a: Right ulna at midshaft in anterior and posterior view, with cross-section in center, b: Right distal femur in posterior view with arrows pointing to sequestra, c: Sacrum and coccyx showing porosity, d: Unsided fibula showing reactive longitudinal periosteal lesions, e: Left distal foot phalanx, ray 1, in dorsal view, showing porosity f: Left fifth metatarsal in dorsal view, showing periosteal new bone growth, g: Left humerus at midshaft (broken postmortem), showing longitudinal fissures and periosteal new bone growth. All scale bars are 1cm.

In addition to this congenital defect, Individual 2 had widespread pathological lesions throughout the cranial and postcranial skeleton. In addition to extremely marked meningeal grooves on the endocranial surface, roughly 52% of the individual’s postcranial skeleton was affected by periosteal lesions, bone sequestra, vascular sclerosis, and porosity (Fig 9). These lesions often resulted in longitudinal sequestra on the outer cortex of bone (such as in Fig 9a, 9b, 9d and 9g), but in the case of the left radius and left femur, the outer-most cortex appeared to detach from the inner-most cortex. Epiphysis preservation was poor, so we are unable to see if the infection localized around the joints. The porotic appearance of bone was observed as superior as the thoracic vertebrae, and as inferior as the metatarsals.

**Fig 10:**
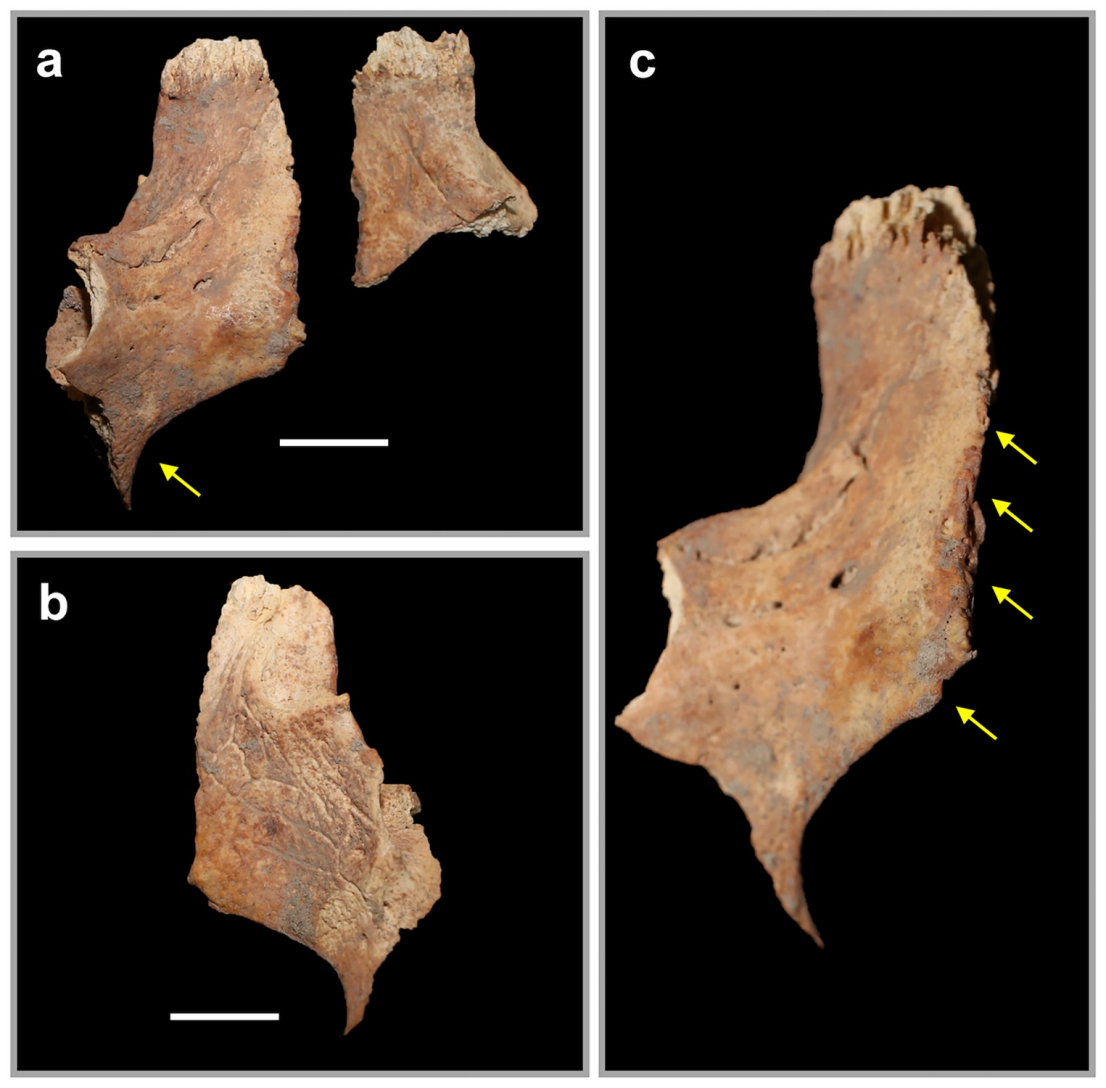
The eroded maxillae of Individual 2. a: Right and left maxillae in anterior view with arrow pointing to widened nasal aperture. b: Right maxilla in medial view. c: Right maxilla in anterior view with arrow pointing towards eroded nasomaxillary border. Scale bars are 1cm.

Notably, the nasomaxillary sutures of both Individual 2’s maxillae appeared eroded and remodeled (Fig 10). The borders themselves were discolored, with the adjacent surfaces smoothed and slightly porous. The lateral margins of the nasal aperture of the maxillae were thin and sharp, with the aperture itself appearing widened.

## Differential diagnoses

### Differential diagnosis of congenital conditions

The complex skeletal morphologies of the brothers in Tomb 45 suggest the presence of similar developmental conditions. For Individual 1, the co-occurrence of two developmental anomalies—the persistent metopic suture and the fourth molar—suggest an interplay of developmental disruptions that are consistent with a congenital syndromic condition, as both develop under the same signaling pathways [41, 42]. While metopic sutures can be non-syndromic, a persistent metopic suture has been known to correlate with certain disorders, including Down’s syndrome [43, 44] and Cleidocranial Dysplasia [43]. Additionally, fourth molars are observed most frequently in individuals with a syndrome [42, 45]. Syndromes that include supernumerary teeth include Cleidocranial Dysplasia, Familial Adenomatous Polyposis, Trichorhinophalangeal syndrome, Type I; Rubinstein-Taybi syndrome; Nance-Horan syndrome; Opitz BBB/G syndrome; Oculofaciocardiodental syndrome; and autosomal dominant Robinow syndrome [46]. Thus, a possible explanation for both congenital traits is Cleidocranial Dysplasia (CCD), a rare autosomal dominant skeletal disease. Present in individuals with supernumerary teeth and metopic sutures, CCD is typically also associated with hypoplastic or aplastic clavicles [47–49]. While Individual 1 does not have any reduction of their clavicles, recent studies have shown the presence of CCD in individuals with morphologically typical clavicles (not hypoplastic or aplastic) [50, 51], allowing for its inclusion as a possible explanation for their skeletal appearance.

Individual 2 also exhibits a developmental interruption—though it is limited to the impacted eruption of their second maxillary molar which became an odontogenic cyst in the crypts of the maxilla. Given that this is the only observed developmental anomaly, it is less conclusive if Individual 2 shared the same or similar condition as Individual 1. Nonetheless, we can deduce that the shared genotypes of both brothers predisposed them to dental interruptions during development.

### Differential diagnosis of acquired diseases

The diffuse bilateral lesions on both Individuals 1 and 2 (Table 1) reflects sustained infectious disease. Additionally, the nonspecific nature of the lesions reflecting infectious disease makes the differential diagnosis difficult and imprecise. Infections that have been found in correlation with bilateral, extensive periosteal lesions and increased skeletal porosity such as those exhibited in Individuals 1 and 2 include osteomyelitis, treponemal disease, tuberculosis, and leprosy. Osteomyelitis, an infection of bone and marrow, is most frequently characterized by destructive bone sequestra, usually localized in long bone metaphyses, and occasionally manifesting on the pelvis, calcaneus and vertebrae [29, 52–54]. Treponemal disease, specifically acquired syphilis, is another possible explanation, as it is characterized by widespread bilateral periosteal lesions and can also include the destruction of the nasal aperture [55]. However, several typical lesions, such as “caries sicca”, are absent in Tomb 45, and the nature of their lesions is less plaque-like than those typical in acquired syphilis [55]. Tuberculosis with skeletal involvement can affect long bone shafts and bones of the hand, but most commonly it affects the spine and major weight-bearing joints (knee, hip and elbow) [56], which is not represented in either Individual 1 or Individual 2. However, lesions on bone shafts, alongside a lack of joint lesions is attested in hypertrophic pulmonary osteoarthropathy (HPOA), which is secondary to tuberculosis [29].

**Table 1:** Locations of periosteal and porous lesions on Individuals 1 and 2.

*Element(s)*	*Individual 1*	*Individual 2*
Cranium	+	-
Mandible	-	-
Hyoid	-	-
Vertebrae	-	+
Clavicle	++	+
Scapula	++	++
Humerus	+	++
Radius	-	++
Ulna	+	++
Carpals	-	-
Metacarpals	-	-
Hand Phalanges	-	+
*Os Coxa*	+	++
Sacrum	+	+
Coccyx	-	+
Femur	-	+
Patella	+	x
Tibia	-	+
Fibula	-	++
Tarsals	+	+
Metatarsals	-	+
Foot Phalanges	+	-

+ = present on one side or on unpaired element

++ = present bilaterally on paired elements

- = no presence

x = not recovered

Additionally, we include in our differential diagnosis leprosy (Hansen’s disease), an infectious disease caused by the bacteria *Mycobacterium leprae*, which can remain progressively active for decades of an individual’s life [29]. Leprosy has been known to spread within family units, not only because transmission is predicated on proximity, but also because one’s susceptibility to leprosy is in part influenced by one’s genetic landscape [57–59]. Further, adolescents between 10 and 19 years are the most susceptible to acquiring leprosy, and males are twice as likely than females to become infected [57, 60].

It is notoriously difficult to identify leprosy in archaeological, fragmented skeletal remains due to the nonspecific nature of many of the lesions [61]. Leprosy is commonly associated with three osteological traits: 1) rhino-maxillary resorption, 2) resorption of the metatarsals and digits, and 3) periosteal lesions on the tibia and fibula [62–65]. Additionally, it can manifest as bilateral “cortical inflammatory changes of the palatine process of the maxilla, diaphyseal cortical surface, and intra-articular cortical surface” and “inflammatory pitting with subsequent subperiosteal new bone formation” [52]. And although leprosy is typically thought of as resulting in the degeneration of the digits, particularly of the hand, it is not necessary for a positive leprosy diagnosis [66]. In bioarchaeological cases, dental calculus and poor oral health have also often been observed in individuals with advanced leprous bone lesions, explained as occurring secondary to decreased ability to chew, practice dental hygiene, and breathe through the nose [61].

While it is possible that the brothers may not have contracted the exact same infectious disease, the patterns of pathological lesions are exceedingly similar between them. These two adult males shared not only a genetic profile, but would have also theoretically shared a living space (broadly defined), making the acquisition of the same infectious disease probable. Lesions are most prolific on Individual 2, with much of the appendicular skeleton exhibiting a porotic appearance and several bones displaying separation of the outer cortex. Signs consistent with leprosy include the degradation of the nasomaxillary region of both maxillae. Yet this is not definitive. The anterior nasal spine, anterior maxilla, and nasal bones were not recovered, therefore preventing a conclusive assessment of the rhinomaxillary region. Further, there is no clear destruction of the digits of the hand or feet, which we may expect to see if leprosy-positive.

The lesions of Individual 1 are less extensive throughout the body than those of Individual 2 (Table 1), which may indicate that the infection was either less progressed or simply had a different manifestation. Outside of the healed traumatic break of Individual 1’s nose, the rhinomaxillary region is unaltered. There is diffuse porosity on the cranial vault, hard palate, and postcrania, a receding maxillary alveolus and remodeled distal fifth metatarsals which feature impressions on their articular surface. The appearance of these metatarsals may be related to early stage leprosy, as the bones were in the process of “pencilling” [67]. With exception of this, the other lesions could be explained by several nonspecific diseases, such as porotic hyperostosis, periostitis, or periodontal disease. Even so, it is the coexistence of several forms of pathological lesions distributed throughout the body that indicates the presence of a severe and chronic infectious disease, whether that be leprosy or not.

## Discussion

Through ancient DNA analysis, the two adult males in Tomb 45 were determined to be brothers. The shared epigenetic landscape may have predisposed them to similar types of disorders and illnesses, with the osteological morphologies supporting a differential diagnosis that includes osteomyelitis, treponemal disease or leprosy. As differential diagnoses are, by nature, open discussions of the many possible etiologies that can result in the lesions described here, we at present cannot identify a single underlying illness without further biomolecular testing. Regardless of the diagnosis, the extensive periosteal and osteolytic lesions, especially those on joint surfaces, may have affected the overlying soft tissue and, by extension, mobility of the two individuals, thereby making certain routine movements or activities difficult. The developed nature of the lesions indicates that they were able to survive with the infection minimally for several years before death. Additionally, when contextualized against contemporary middle-status groups at Megiddo, we assert that the elite individuals in Tomb 45 were relatively well-provisioned during life as evidenced through their survival despite possible congenital anomalies and infectious diseases. At Megiddo in both the Middle and Late Bronze Ages, individuals from the less prosperous middle-status areas show no severe pathological afflictions beyond osteoarthritis, LEH, and periodontal disease [68–71]. In contrast, the elite individuals presented here from Tomb 45, as well as those in the royal Tomb 50 show higher frequencies of lesions associated with long-endured disorders and infectious diseases [9]. This is not to say that these illnesses were not also present in the middle-status groups, but without the same resources as the elite, those afflicted may not have been able to survive long enough for their bones to reflect it [72].

The presence of a trephination on Individual 1 further represents an unusual and high-level intervention that indicates access to the services of a trained practitioner who administered this treatment shortly before death. This trephination thus illuminates the intersection of biological circumstance and social action in antiquity. Infrequently observed in the archaeological record, there are only a few dozen trephinations recorded in the entire history of the Levant [33, 73–76]. The trephination style of the Megiddo individual, defined as angular-notched, is even less common, only attested in four other southern Levantine cases: one from Iron Age Timna (modern Israel) [73] and three from Iron Age Lachish (modern Israel) [25, 77–79], all of which were performed on males and all of whom appeared to have experienced no postoperative healing. Thus, Megiddo’s case is the earliest of its kind in the region by at least several centuries. It is also the only example of the five that was found alongside the excised cranial fragments.

Overall, trephinations have proven medicinal benefits [80, 81], and are most often cited in the archaeological literature as curative to head trauma by releasing pressure buildup in the head [82]. And while these procedures are less frequently linked to curing non-traumatic illnesses and diseases, they have been performed on individuals who had epilepsy, scurvy, frontal sinusitis, intracranial disorders and diseases, hydrocephaly and osteitis [74, 75, 83, 84]. However, there are no archaeological examples of trephination on an individual exhibiting diffuse, extensive lesions as is the case in our study.

The further inclusion of the excised cranial pieces with Individual 1 suggests their intentional re-placement with the individual, though it remains unclear why. One possibility is that they were placed in the trephined hole post-operation to reduce the hole’s size and encourage osseous healing. In clinical literature, examples of bone re-insertion (autologous cranioplasty) are well documented, albeit with mixed results as to whether it indeed encourages healing [85]. The replacement of bone in an empty space essentially acts as a physical scaffold, which is thought to facilitate remodeling after the removal of bone [86]. However, while it has been found as being restorative for hole closure [34, 79], without proper hygienic storage conditions between excision and replacement, the reinsertion of the bone may lead to severe complications [87].

Some examples of this phenomenon of bone reinsertion also exist in the archaeological literature, though none show signs of postoperative healing. In Inca Peru (c. 1400–1532 CE), an angular notched trephination (also referred to as linear cutting, crosscut sawing, or rectangular intersecting incisions) was performed on an older female, and a square piece of excised bone was reinserted into the wound, with organic matter placed on top [88, 89]. Similarly, an individual from Crichel Down Dorset dating to the Bell Beaker Period (c. 2800–1800 BC) had an excised roundel reinserted into the trephination opening [90]. It is possible that more there were occurrences of this method in the past, but that excised bone is lost during post-depositional and excavation processes. Additionally, while clinical literature suggests that osseous healing does not minimally begin until several weeks post operation, it is unclear whether ancient practitioners believed this technique would facilitate the healing process. In short, what we understand today about the risks and rewards of the reinsertion of trephined bone was likely different than that of the practitioners in antiquity. Nevertheless, the cranial trephination on Individual 1 presents an avenue for future interrogation of the cross section of illness and treatment in the archaeological record. When paired with the pathological appearance of much of Individual 1’s remains, we propose that this operation was meant as an intervention to deteriorating health, but was ultimately unsuccessful.

To close, we wish to highlight that the brothers of Tomb 45, despite their pathological state, were inhumed in a typical custom for the period with fine offerings. They were, importantly, not “othered” or separated from the traditions that characterized their contemporaries in the city. Rather, they were therefore provisioned for in life and death in a way that highlights the particular dynamics amongst a portion of this elite family in Late Bronze Age Megiddo.

## Supporting information

S1 Fig Excavation sequence of Individual 2. (TIF)

S2 Fig Articulated foot of Individual 2. Individual 1 is greyed out to show boundaries of Individual 2.
(TIF)

S3 Fig Archaeological context of Individual 1. Location of white bead on cranium shown in the red box. Hatched square shows in situ location of vessels shown in the yellow box.
(TIF)

S4 Fig Additional views of the cranial trephination and excised cranial pieces. (TIF)

S5 Fig Additional views of the fifth metatarsals of Individual 1. (TIF)

S1 Text Burial sequence and context of Tomb 45. (DOCX)
