# Supplementary figures and images for: Cranial trephination and infectious disease in the Eastern Mediterranean: The evidence from two elite brothers from Late Bronze Megiddo, Israel
Source: PLoS One. 2023 Feb 22;18(2):e0281020. doi: 10.1371/journal.pone.0281020 (PMC9946252; doi:10.1371/journal.pone.0281020)

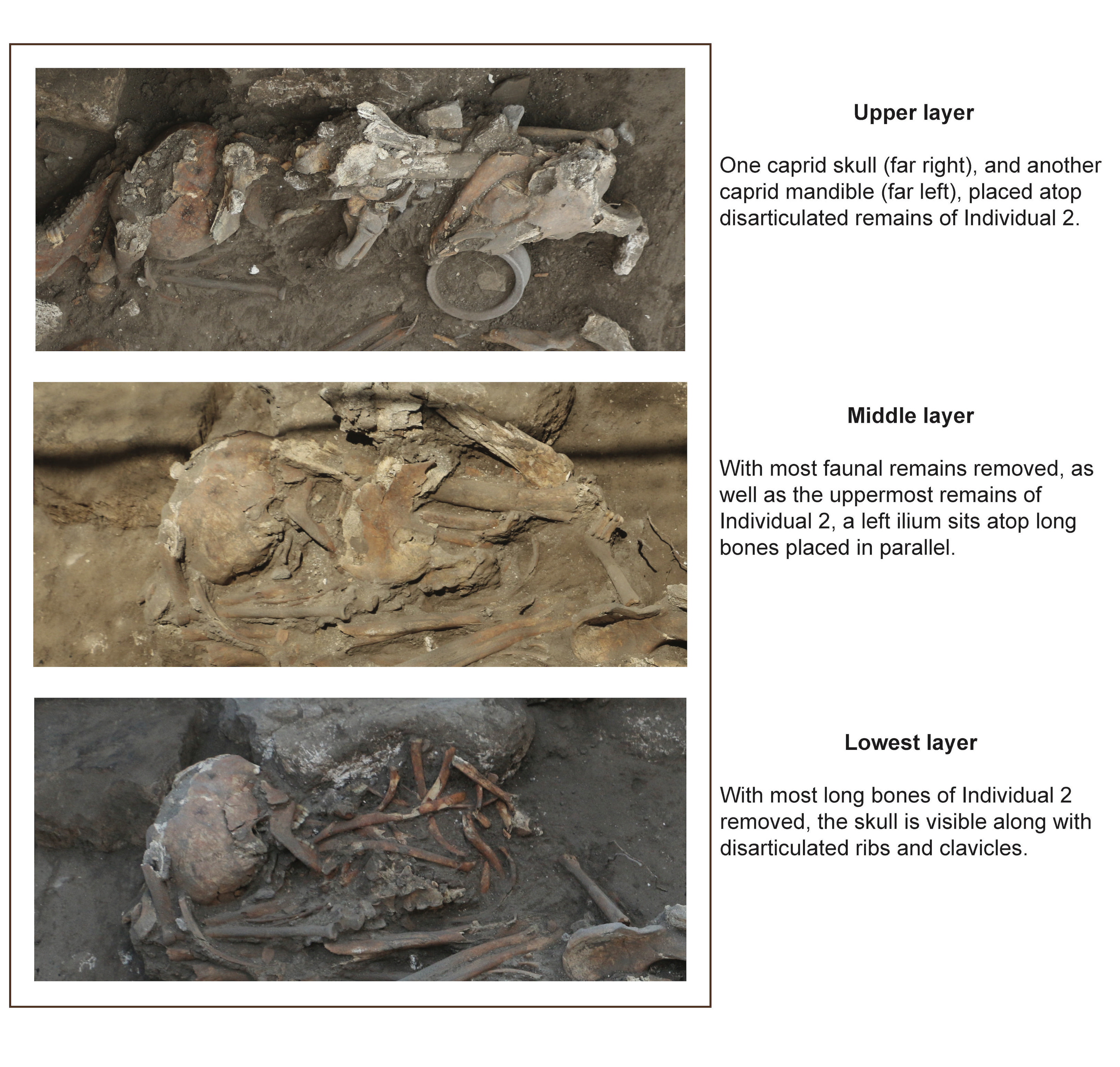

Supplement: S1 Fig — (TIF) [file pone.0281020.s001.tif]

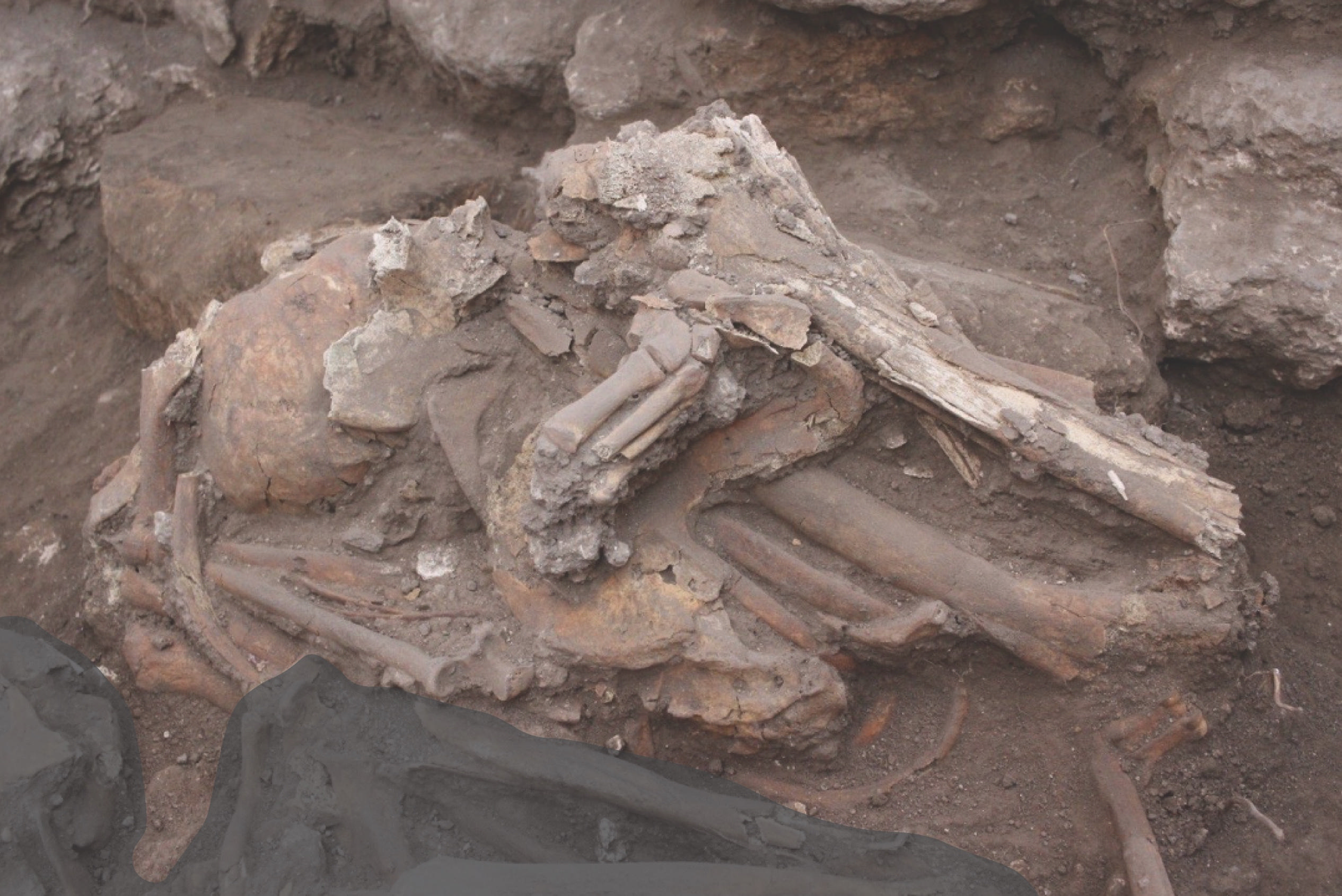

Supplement: S2 Fig — Individual 1 is greyed out to show boundaries of Individual 2. (TIF) [file pone.0281020.s002.tif]

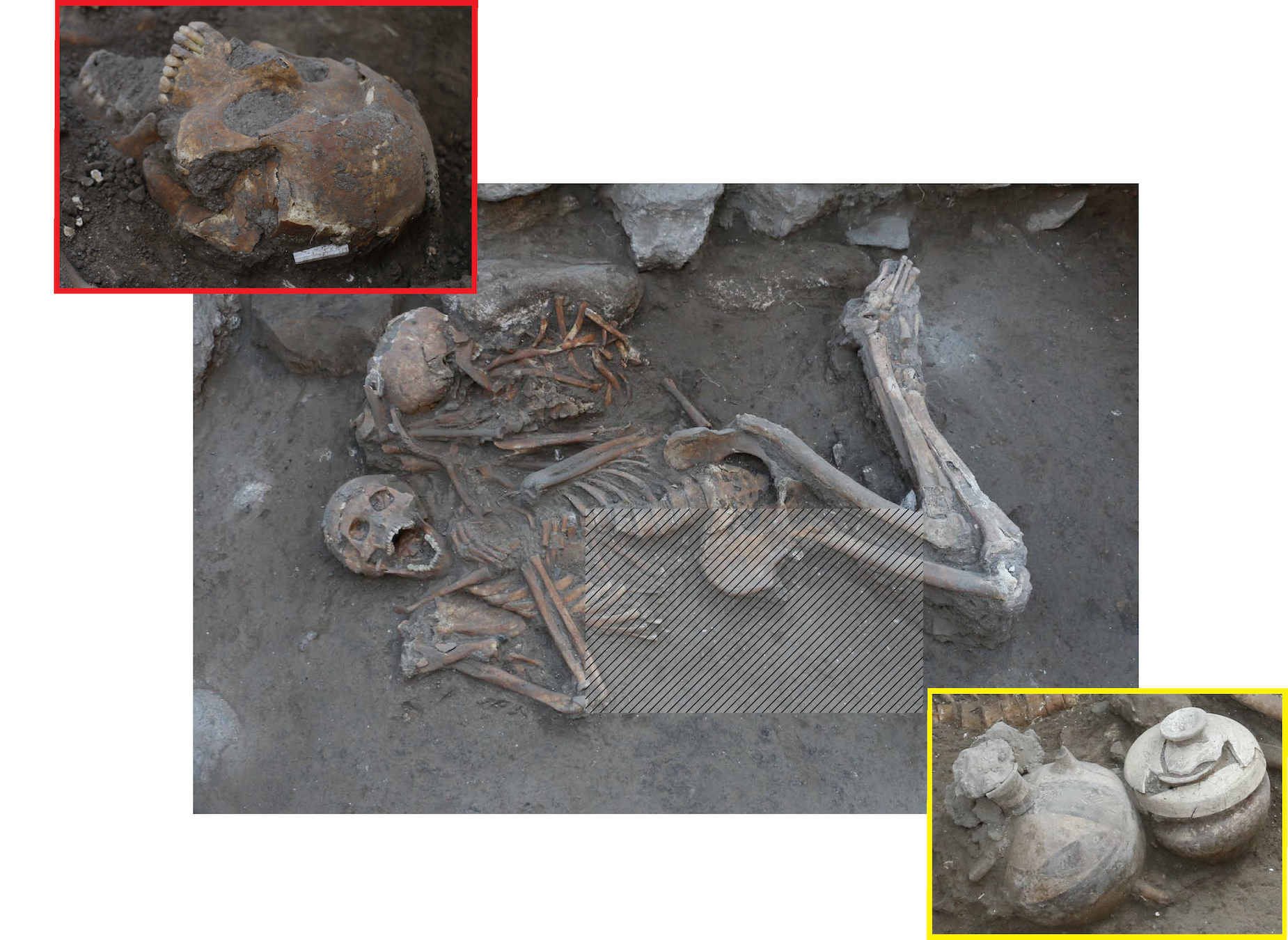

Supplement: S3 Fig — Location of white bead on cranium shown in the red box. Hatched square shows in situ location of vessels shown in the yellow box. (TIF) [file pone.0281020.s003.tif]

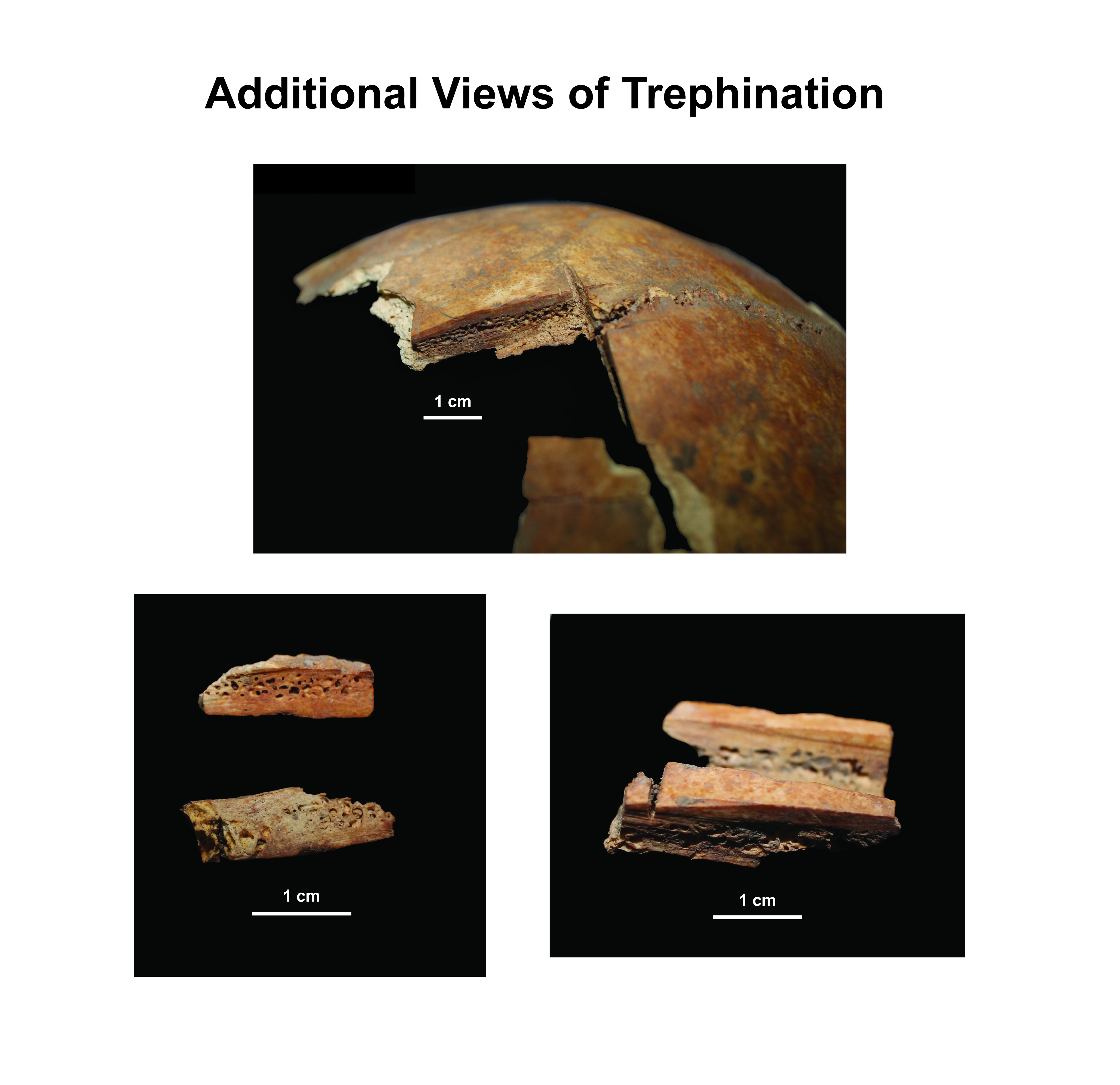

Supplement: S4 Fig — (TIF) [file pone.0281020.s004.tif]

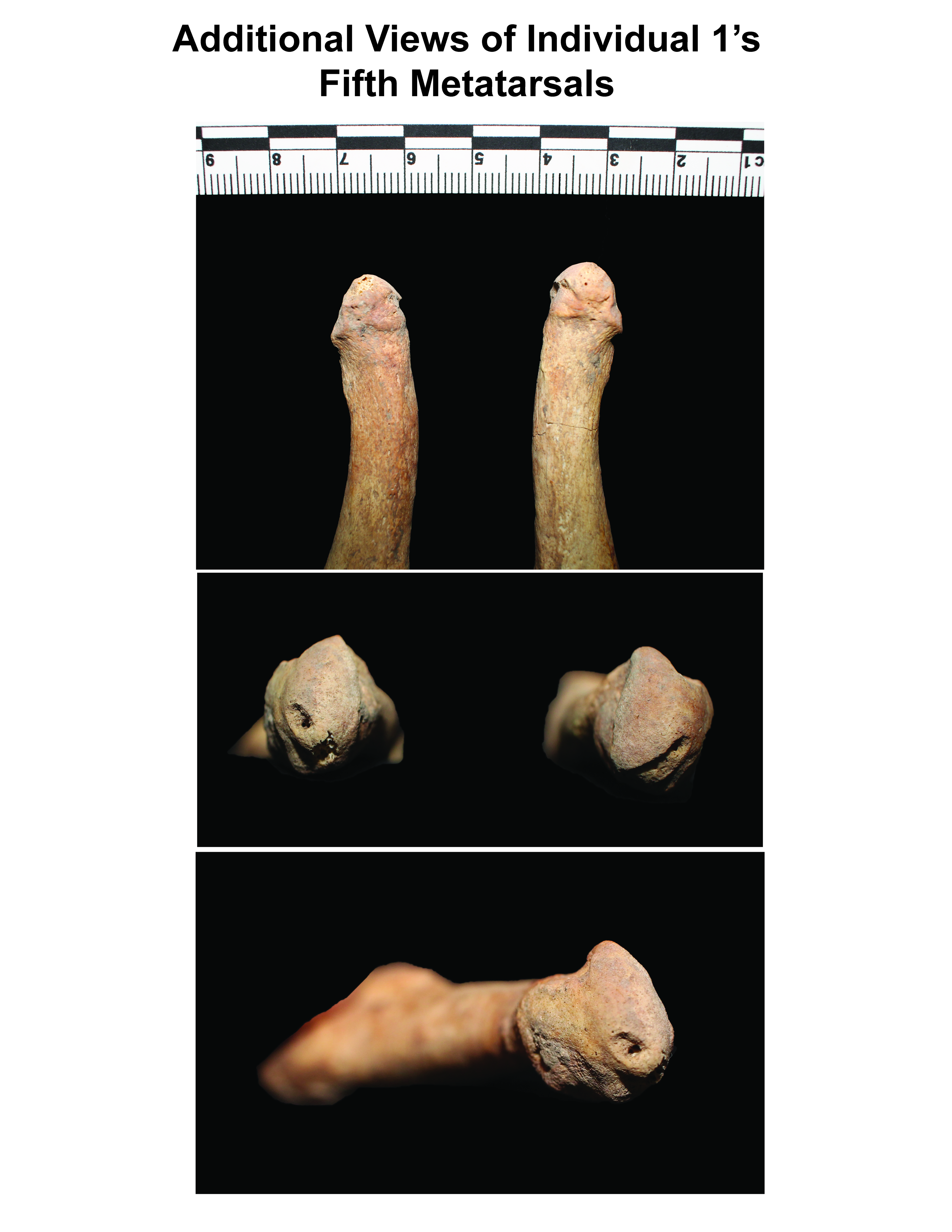

Supplement: S5 Fig — (TIF) [file pone.0281020.s005.tif]
