# Supplementary material for: Cranial trephination and infectious disease in the Eastern Mediterranean: The evidence from two elite brothers from Late Bronze Megiddo, Israel
Source: PLoS One. 2023 Feb 22;18(2):e0281020. doi: 10.1371/journal.pone.0281020 (PMC9946252; doi:10.1371/journal.pone.0281020)
Supplement: S1 Text — (DOCX) [file pone.0281020.s006.docx]

**S1 Text: Burial Sequence and Context of Tomb 45**

**Phase I: Burial of Individual 2**

The first individual to die and be buried was Individual 2. This is evidenced by their remains having already been mostly skeletonized at the time of burial for Individual 1. When exhumed, the remains were collected and repositioned at the periphery of the burial. Based on the layers of the skeletal deposit, the sequence of gathering the bones indicates that the head and axial skeleton were placed on the bottom, and the larger bones of the limbs and pelvis were placed in an organized, parallel bundle on top. The decomposed remains of two caprids and a cooking pot were placed on top of the entirety of Individual 2. The excavation of this individual proceeded in layers (S1 Fig), which reveals the sequence of deposition for the individual elements.

Several joint articulations remained intact, namely the temporomandibular joint and the bones of the left foot (S2 Fig). The bones of the left foot are classified as labile joints [1,2]. Labile joints decompose relatively quickly, compared to persistent joints which take much longer [2]. Although a myriad of intrinsic and extrinsic factors influence joint breakdown [3–6], the fact that labile joints were at least partially intact when the body of Individual 2 was disturbed suggests that they were mostly, but not fully, skeletonized when exhumed [7]. This allows us to consider the rate of decay of Individual 2, which speaks to the period that could have elapsed between the two brothers’ deaths. Given the presumed burial context—a shallow, filled dirt pit, containing a shrouded body—alongside the dry summers and wet winters of the Mediterranean, we posit that the burial of Individual 1 occurred relatively shortly (1-3 years) after Individual 2’s death.

**Phase II: Burial of Individual 1**

Individual 1 was inhumed with the torso supine and legs laterally flexed on the right side. Arms were crossed over the chest, with the right hand above the left, each slightly contracted into a fist. The “verticalization” of the clavicles reflects transversal compression of the shoulders [2], which, alongside the flexion of the legs, supports that the body was bound in a shroud prior to burial [8,9]. A white frit or shell bead was found *in situ* on the superior cranium and several vessels, including jugs of Chocolate-on-White Ware and Cypriot Bichrome, which were found by the legs (S3 Fig).

**References**

1. Duday H, Guillon M. Understanding the Circumstances of Decomposition when the Body is Skeletonized. In: Schmitt A, Cunha E, Pinheiro J, editors. Forensic Anthropology and Medicine: Complementary Sciences from Recovery to Cause of Death. Totowa, NJ: Humana Press; 2006. pp. 117–157.

2. Duday H, Cipriani AM, Pearce J. The Archaeology of the Dead: Lectures in Archaeothanatology. Oxbow Books; 2009.

3. Fiedler S, Graw M. Decomposition of buried corpses, with special reference to the formation of adipocere. Naturwissenschaften. 2003;90: 291–300. doi:10.1007/s00114-003-0437-0

4. Pinheiro J. Decay Process of a Cadaver. In: Schmitt A, Cunha E, Pinheiro J, editors. Forensic Anthropology and Medicine. Totowa, NJ: Humana Press; 2006. pp. 85–116. doi:10.1007/978-1-59745-099-7_5

5. Mickleburgh HL. Actualistic Experimental Taphonomy of Inhumation Burial. In: Barone PM, Groen WJM, editors. Multidisciplinary Approaches to Forensic Archaeology: Topics discussed during the European Meetings on Forensic Archaeology (EMFA). Cham: Springer International Publishing; 2018. pp. 105–114. doi:10.1007/978-3-319-94397-8_7

6. Kõrgesaar K, Jordana X, Gallego G, Defez J, Galtés I. Taphonomic model of decomposition. Leg Med. 2022;56: 102031. doi:10.1016/j.legalmed.2022.102031

7. Mickleburgh HL, Wescott DJ. Controlled experimental observations on joint disarticulation and bone displacement of a human body in an open pit: Implications for funerary archaeology. J Archaeol Sci Rep. 2018;20: 158–167. doi:10.1016/j.jasrep.2018.04.022

8. Brody AJ. Late Bronze Age Intramural Tombs. In: Stager LE, Schloen DJ, Master DM, editors. Ashkelon I: Introduction and Overview (1985-2006). Winona Lake, IN: Eisenbrauns; 2008.

9. Cradic MS. Embodiments of death: The funerary sequence and commemoration in the Bronze Age Levant. Bull Am Sch Orient Res. 2017;377: 219–248.
